# Human Mobility in a Continuum Approach

**DOI:** 10.1371/journal.pone.0060069

**Published:** 2013-03-28

**Authors:** Filippo Simini, Amos Maritan, Zoltán Néda

**Affiliations:** 1 Center for Complex Network Research and Department of Physics, Biology and Computer Science, Northeastern University, Boston, Massachusetts, United States of America; 2 Institute of Physics, Budapest University of Technology and Economics, Budapest, Hungary; 3 Dipartimento di Fisica e Astronomia “G. Galilei”, Università di Padova, CNISM and INFN, Padova, Italy; 4 Department of Physics, Babeş-Bolyai University, Cluj-Napoca, Romania; Semmelweis University, Hungary

## Abstract

Human mobility is investigated using a continuum approach that allows to calculate the probability to observe a trip to any arbitrary region, and the fluxes between any two regions. The considered description offers a general and unified framework, in which previously proposed mobility models like the gravity model, the intervening opportunities model, and the recently introduced radiation model are naturally resulting as special cases. A new form of radiation model is derived and its validity is investigated using observational data offered by commuting trips obtained from the United States census data set, and the mobility fluxes extracted from mobile phone data collected in a western European country. The new modeling paradigm offered by this description suggests that the complex topological features observed in large mobility and transportation networks may be the result of a simple stochastic process taking place on an inhomogeneous landscape.

## Introduction

Human mobility in form of migration or commuting becomes increasingly important nowadays due to many obvious reasons [1]: (i) traveling becomes easier, quicker and more affordable; (ii) some borders (like the ones inside EU) are more transparent or even inexistent for travelers; (iii) the density and growth of the population and their gross national product presents large territorial inequalities, which naturally induces mobility; (iv) the main and successful employers concentrate their location in narrow geographic regions where living costs are high, hence even in developed countries the employees are forced to commute; (v) large cities grow with higher rates, optimizing their functional efficiency and creating the necessary intellectual and economic surplus for sustaining this growth [2]. This higher growth rate of the population can be achieved only by relocating the highly skilled work-force from smaller cities. Here we propose a unified continuum approach to explain the resulting mobility patterns.

Understanding and modeling the general patterns of human mobility is a long-standing problem in sociology and human geography with obvious impact on business and the economy [3]. Research in this area got new perspectives, arousing the interest of physicists [4], [5] due to the availability of several accurate and large scale electronic data, which helps track the mobility fluxes [6]–[8] and check the hypotheses and results of different models. Traditionally mobility fluxes were described by models originating from physics. The best-known is the *gravity model* [6], [9] that postulates fluxes in analogy with the Newton’s law of gravitation, where the number of commuters between two locations is proportional to their populations (i.e. the ‘demographic mass’) and decays with the square of the distance between them. Beside the well-known gravity model, several other models were used like the generalized potential model [10], [11], the intervening opportunities model [12] or the random utility model [13]. Recently, a parameter-free *radiation model* has been proposed, leading to mobility patterns in good agreement with the empirical observations [14]. The model was developed assuming a spatially discretized settlement structure, and consequently it operates with a discretized flux topology on the edges of a complete graph. Here we consider and test a continuum approach to this model operating with fluxes between any two regions, and show that several other mobility models can be derived within the same framework. This novel approach based on the continuum description offers a new modeling and data interpretation paradigm for understanding human mobility patterns.

## Results

### The Modeling Framework

The radiation model [14] has been originally formulated to estimate commuting fluxes, i.e. the average number of commuters traveling per unit time between any two locations in a country. The key idea is that while the home-to-work trip is a daily process, it is determined by a one-time choice, i.e. the job selection. Therefore commuting fluxes reflect the human behavior in the choice of the employment. In real life many variables can affect the employment’s choice, from personal aspirations to economic considerations, but for the sake of simplicity only the most influential variables are considered in the model: the salary a job pays (or more generally, the working conditions), and the distance between the job and home. The main idea behind the model is that an individual accepts the closest job with better pay: *each individual travels to the nearest location where she/he can improve her/his current working conditions (benefits)*. With this assumption, the probability 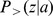 that an individual with benefit 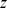 refuses the closest 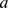 offers is:
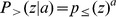
(1)


where 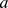 is the number of open positions in the area within a circle of radius 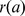 centered in the origin location, and 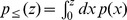 is the cumulative distribution function of the benefits. Equation (1) is equivalent with assuming that the rejection of 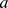 job offers with benefits less or equal to 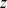 are independent events.

Making different assumptions and approximations on the benefit distribution 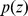, one can obtain several formulas for the number of trips between locations. Below we present four examples: the *original radiation model*, the classic *intervening opportunities* (IO) model [12], a *uniform selection model*, and a novel *radiation model with selection*.

#### The original radiation model

If we solve Eq. (1) assuming that the benefit distribution 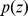 is a continuous function, we recover the original radiation model’s formula [14]. Indeed, we calculate the probability 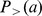 of not accepting one of the closest 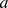 job offers by integrating Eq. (1) over the benefits:
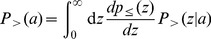
(2)

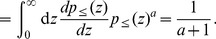
(3)


#### The intervening opportunities model

We can also show how the classical IO model [12], [15] can be included within the same framework as a degenerate case. Consider the situation in which the benefit distribution is singular, i.e. all jobs are exactly equivalent 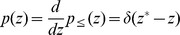 and 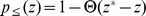 (where 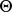 is the Heaviside function). In this case we have to specify the individual’s behavior when s/he receives a job offer identical to her/his current one: this corresponds to setting a specific value to the step function at the discontinuity point, 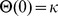. If 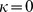, then the individual will travel to an infinite distance; while if 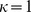, the individual accepts the job in the closest location. If 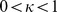, then the individual accepts each offer with probability 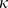 and refuses it with probability 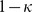. Applying Eq. (2) we obtain
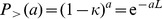
(4)


where 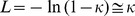 if 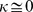.

#### The uniform selection model

When 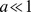, a good approximation of Eq. (4) is 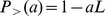, which corresponds to randomly select one of the available job opportunities, irrespective of the benefits and the distance. Generalizing this interpretation, we can define a model on a finite space containing 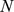 average job openings per unit time in which the accepted job is selected uniformly at random, and thus 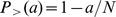.

#### The radiation model with selection

Let us assume that the benefit distribution 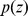 is continuous as in the original radiation model, whereas the probability to accept any offer is reduced by a factor 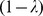 with 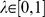. As a consequence, the probability that an individual with benefit 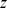 accepts an offer has to be replaced by a reduced value: 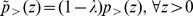. This process can be interpreted as a commuting population who is willing to accept better offers with probability 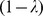, or who is aware only of a fraction 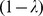 of the available job offers. This is equivalent to a combination of the radiation model and the intervening opportunities model described above (here 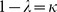). In this case 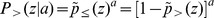, and the probability to refuse the closest 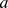 offers is
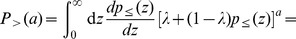


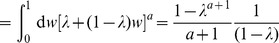
(5)


Note that when 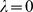 we recover the original radiation model (3), while a 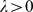 causes a shift of the median of 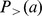 towards higher values of 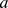. In particular, for 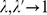 the following approximation holds: 
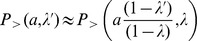
, where we made explicit the dependence on 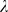. The validity of this relationship can be verified by defining 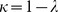 and expanding around 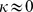:
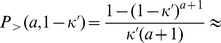


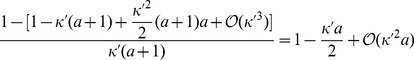
(6)


and
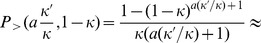


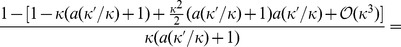


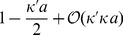
(7)


The difference is of the order 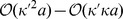, thus 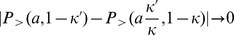 when 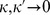. Note that Eq. (7) follows immediately from Eq. (6) by substituting 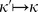 and 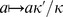. We can derive the dependence of the median on the rescaling of the parameter 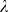: if with 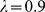 the median is 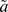 defined by 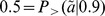, with 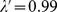 the median is ten times higher, i.e. 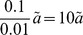. By varying the parameter 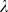 it is thus possible to adjust the median of the distribution 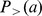, which is equivalent to set a characteristic length of the trips.

These examples show the versatility of the radiation model’s formalism, which can successfully provide an explanation to several probability distributions 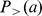 observed empirically in different contexts [12], [14]. The probability density, 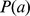, to accept one of the offers between 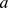 and 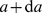 for a unit 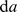 value can be obtained from 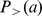 by derivation. To be more specific, let us consider the original radiation model. From Eq. 3 we have 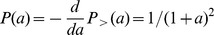. Let 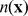 be the density of job offers at point 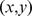 (in polar coordinate, 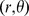, we will use the same notation for the density 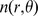). Then one gets the following expression for the number of job offers within a distance 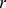 from 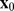, 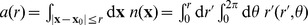 and 
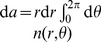
. Thus the probability to accept an offer within a region at distance between 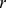 and 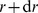, 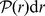, is given by
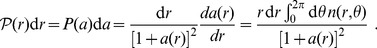
(8)


**Figure 1:**
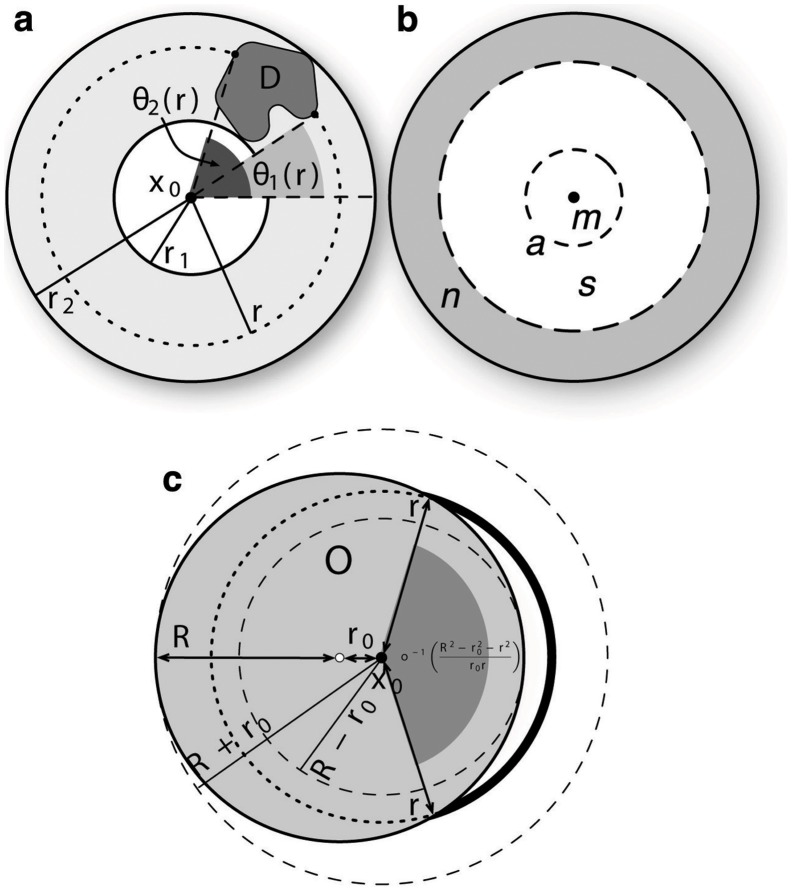
Definition of the variables used in the calculations. a) Notation used in Eq. 10. b) Configuration used to calculate the probability 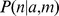 c) Configuration used in Eq. (12) to calculate 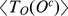.

This also suggests that
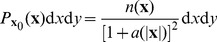
(9)


is the probability to travel from the origin, 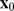, to an area 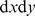 centered at the spatial point 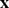. In general, 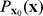 has the following simple expression for any model presented above: 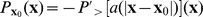. From Eq. (8) we can derive the probability 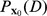 of a trip from the origin to a generic region 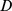 (see Fig. 1a) as
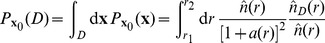
(10)


where 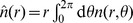 is the radial job offers’ density, and 
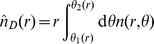
 is the job offers’ density in 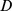 at distance 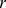 from 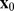. If the radial job offers’ density has small variations around its average between 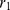 and 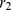, i.e. 
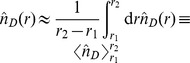
 and 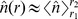 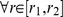, then we can derive a simple approximated formula for 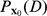


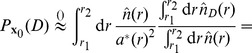


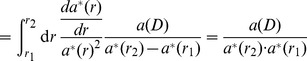
(11)


where 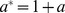, and 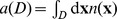 is the number of job offers in 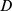.

This equation is especially important because data are usually collected as fluxes in a discretized space, whose regions are defined according to the local administrative subdivision (e.g. counties or municipalities). 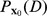 has a particularly simple expression if we consider the probability 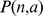 to accept one of the 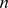 offers between 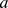 and 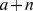, corresponding to the ring in Fig. 1b. This is given by 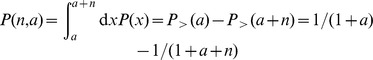, which in the limit 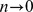 tends to 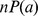. If we only consider trips outside a circular region centered on the origin location and containing 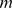 job offers, then the probability 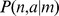 to accept one of the 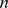 offers between 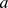 and 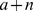 given that none of the closest 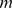 offers has been accepted, is 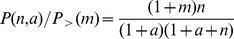. Note that 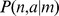 is the same probability of one trip derived in the original radiation model’s discrete formulation [14] with the only difference being that here we have 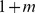 instead of 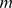 (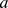 is equal to 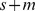).

It is important to observe that the equations derived for 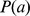 are correctly normalized when the total number of job offers, 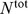, is infinite and therefore finite-size corrections are required in real-world applications [16]. The normalized probability is 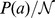, where the normalization constant is 
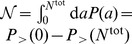
. The correction to 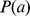 is of the order 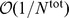, which in most cases is very small given that usually 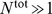. This normalization scheme has a straightforward mechanistic interpretation: it offers another try at job selection for individuals who during their first job search did not find any job offer with better benefit than their current one. Other kinds of normalization procedures that combine two of the models presented above are also possible. If, for example, we assume that the individuals who did not find a better job in their first try decide to select the offer with the highest benefit, even if it does not exceed their current one, (a mechanism corresponding to the random selection model) the normalized probability we obtain is 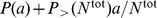. Therefore, there are multiple ways to normalize the models, each capturing a different selection mechanism. This suggests that a systematic investigation of finite size effects could also help understand the mechanisms underlying job selection.

### Comparison with Empirical Data

**Figure 2:**
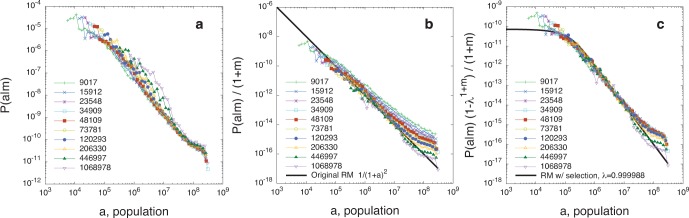
Testing the radiation model’s theoretical predictions on commuting trips extracted from the US census dataset. *a*) We divide the commuting flows in deciles according to the population of the origin county, 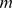, and for each set we calculate the distributions 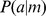. The values in the key indicate the mean origin population, 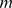, of each decile. We use the population as a proxy to estimate the number of employment opportunities in every county, 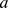, assuming in first approximation a linear relationship between population and job openings. *b*,*c*) The collapse of the distributions 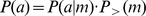 on the theoretical curves Eqs. (3) and (5) predicted by the original radiation model and the radiation model with selection respectively. (See the section *Materials and Methods* for details).

**Figure 3:**
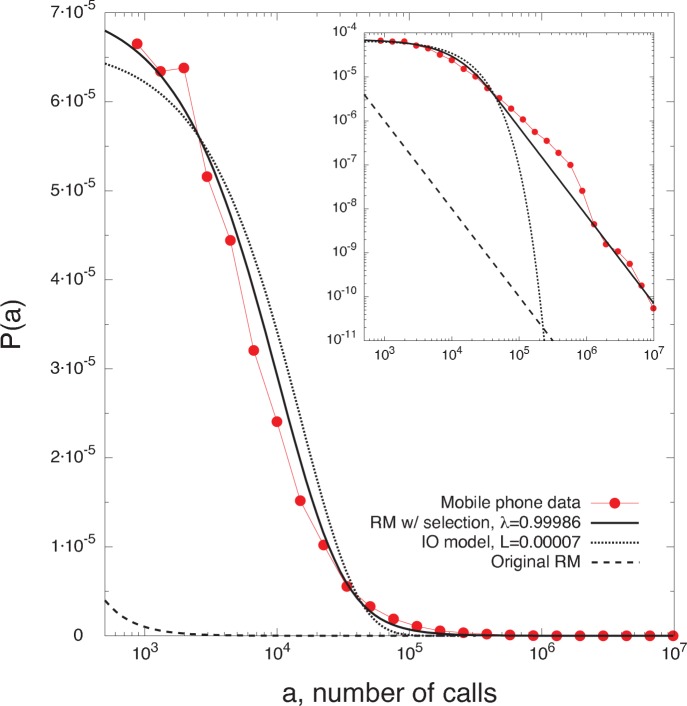
Testing the mobility models on trips extracted from a mobile phone dataset. We analyze all call records collected during one day, and we define a trip when we observe two consecutive calls by the same user from two different towers. We define the variable 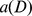, representing the number of possible points of interest in a circular area 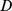 centered at a given cell tower, as the total number of calls placed from the towers in 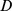, assuming that a location’s attractiveness is proportional to its call activity. We then calculate the empirical distribution 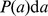, i.e. the fraction of trips to the towers between 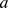 and 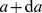 (red circles), and we compare it to the various models’ theoretical predictions 
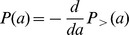
, with 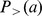 defined in Eqs. (??), (4), and (3), and whose parameters, 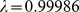 and 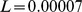, are obtained with least-squares fits (black lines). In the inset we show the plot in a log-log scale. (See the section *Materials and Methods* for details).

In Fig. 2 we apply the original parameter-free radiation model (Eq. 3) and the one-parameter radiation model with selection (Eq. 5) to commuting data among United States’ counties. We show the agreement between the theoretical 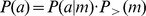 distributions and the collapses predicted by the original radiation model, Fig. 2b, and the radiation model with selection, Fig. 2bc. In Fig. 3 we compare the theoretical distributions 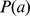 of the original radiation model, the radiation model with selection, and the IO model, to the empirical distributions extracted from a mobile phone database of a western European country. For a description of the data sets and the analyses performed see the section *Materials and Methods*.

An advantage of the proposed approach is that it is defined for a continuous spatial density of job offers, and its results are thus independent of any particular space subdivision in discrete locations. This feature solves some consistency issues present in other mobility models defined on a discretized space. Consider for example the gravity law [6], [9], [17], the prevailing framework to predict population movement [18]–[20], cargo shipping volume [21], inter-city phone calls [22], as well as bilateral trade flows between nations [23]. The gravity law’s probability of one trip from an area with population 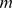 to an area with population 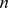 (assuming that population is proportional to the number of job offers) at distance 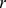 is obtained by fitting a formula like 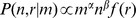 to previous mobility data. As shown in [14], the values of the best-fit parameters 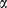 and 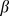 are strongly dependent on the spatial subdivision considered, raising the problem of deciding which subdivision gives the correct results.

Also, the continuous formalism developed here helps finding a solution to the issue concerning the additivity of the fluxes frequently encountered in discrete formulations. As an example, consider two adjacent areas, 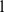 and 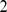 with populations 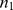 and 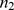 respectively, at the same distance 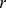 from the origin location. The gravity law predicts 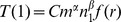 and 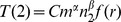 travelers to 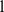 and 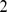 respectively. If we consider a different spatial subdivision, in which locations 1 and 2 are now grouped together forming a single location, 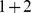, and we calculate the number of travelers we obtain 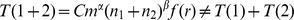 unless 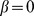 or 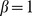. If the exponent 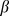 is different from one, the additivity requirement does not hold and the difference in the estimated trips can be considerably high. For example, if 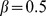 and 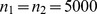, then 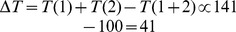, i.e. a 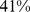 relative difference. The additivity of the fluxes is a necessary property required to any mobility model in order to be self-consistent. We can easily verify that all models derivable from Eq. (1) have the additivity property. This is a consequence of the linearity of the integral in Eq. (10). In fact, for every two regions 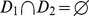 and 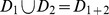 we have 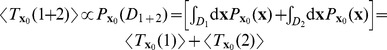, for a generic 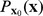. We observe that it is possible to develop a continuum formalism for the gravity model that fulfils the additivity constraint by assuming that the probability to travel from location 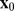 to location 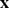 is 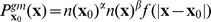. The average number of travelers from region 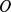 to region 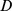 is 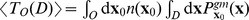 and because of the linearity of the integral on 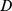 the fluxes are additive.

We can use the continuum approach to investigate the relationship between a region’s population and the total number of travelers from that region outwards (i.e. the commuters whose destination is outside the region). It is often assumed that the number of commuters is proportional to the region’s population. This is the case, for example, for the commuting fluxes measured by the US census 2000 [14]. We can check the validity of this assumption by writing the average number of commuters leaving a region 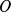 as 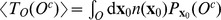, where 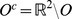 is the complement of 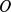, and 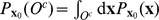 is the probability for an individual in 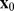 to travel outside 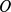 (cf. Eq. 10). We can easily calculate 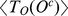 if we make the simplifying assumptions that the number of job offers in a region is proportional to the region’s population (see the section *Materials and Methods* for details), that the population density is uniform, i.e. 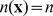, and 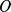 is a circle of radius 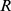 (see Fig. 1c). Then
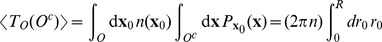


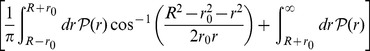
(12)


where 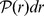 is the probability to travel to a distance 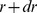 (cf. Eq. 8). For the original radiation model 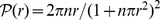, and Eq. (12) can be calculated exactly and has the following asymptotic limits: 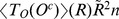 if 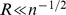, and 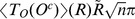 if 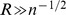. The same asymptotic behaviour is obtained for the IO model, with 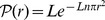: 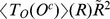 if 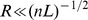, and 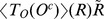 if 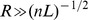. For both models if the size of the region, 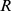, is sufficiently small then the number of commuters, 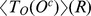, is proportional to the total population of the region. When 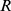 becomes larger than a characteristic size only the individuals living close to the boundary have a non-zero chance of travelling outside 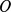.

A further generalization of the model could take into account the fact that Euclidean distance is not appropriate in situations where geographical barriers exist and/or travel facilities are heterogeneously distributed. In this case one introduces a metric tensor 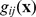 and the square distance between neighboring positions at point 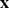 is 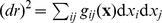 with 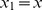 and 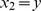. In this case Eq. (9) is rewritten as 
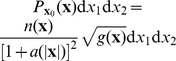
, where 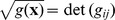 is a local parameter of the model.

## Discussion

The fundamental Eq. (1) represents a unified framework to model mobility and transportation patterns. In particular, we showed how the intervening opportunity model [12] can be regarded as a degenerate case of the radiation model, corresponding to a situation in which the benefit differences are not taken into account in the employment’s choice. We also explained the advantages of a continuous approach to model mobility fluxes, we derived the appropriate discretized expressions that guarantee the consistency of our predictions on any discrete spatial subdivision, verifying that the fluxes additivity requirement holds.

Furthermore, our approach also provides an insight on the theoretical foundation of the most common types of gravity models. Indeed, when the space is homogeneous and the job’s distribution is fractal, 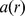 is independent of the point of origin, i.e. 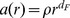 where 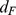 and 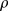 are the fractal dimension and an average density of job offers, respectively. Equation (11) for the probability, 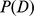, to observe a trip to a generic region 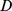 within distances 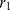 and 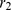 from the origin becomes (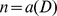 is the number of job offers in D) 
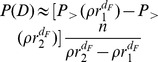
. In particular, for the original radiation model, Eq. (3), the average number of trips to a region 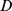 containing 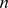 job offers is 
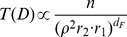
, whereas for the intervening opportunities model, Eq. (4), 
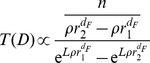
. These two classes of deterrence functions 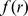, power law and exponential, are actually the two most used form of gravity models [17], [20], [24]. Moreover, our approach provides an interpretation to the gravity model’s fitting parameters. First, the exponents 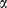 and 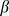 are both one when the benefits are spatially uncorrelated, i.e. the benefit distributions at the local (regional) and global (country) scales are the same. If 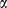 or 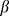 differ from one it means that there are regions where job offerings with higher or lower benefits tend to concentrate. Second, the exponent of the power law is predicted to be two times the fractal dimension of the job offers, 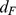, whereas the exponential deterrence function should be substituted with a stretched exponential with shape parameter 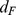 and a characteristic length of the order of 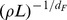. Thus, when the spatial displacement of the potential trip’s destinations is a fractal, the radiation model’s formalism offers a theoretical derivation of the gravity models from first principles.

In conclusion, we have developed a general framework for unifying the theoretical foundation of a broad class of human mobility models. The used continuum approach allows for a consistent description of mobility fluxes between any delimited regions. The successful comparison with real mobility fluxes extracted from two different data sources confirms that our approach not only provides a theoretically sound modeling framework, but also a good quantitative agreement with experimental data. This suggests that the decision process we assumed for the job selection also captures the basic decision mechanism related to the choice of the destinations for other activities (shopping, leisure, …). On the other hand, our study suggests that the weighted network representing the mobility fluxes among geographic regions can be the result of a stochastic process consisting of many independent events. This approach is somehow complementary to the theory of optimal transportation networks [25]–[30] that describes the patterns observed in different natural and artificial systems solely as the adaptation to a global optimization principle (e.g. leaf venations, river networks, power grids, road and airport networks). The modeling framework we propose provides also a plausible example of spontaneous bottom-up design of transportation networks. Indeed, we show how complex patterns can arise even in those systems lacking a global control on the network topology, or a long-term evolutionary selection mechanism of the optimal structure.

## Materials and Methods

### Analysis on the Inter-county Commuting Trips Extracted from United States’ Census Data

The data on US commuting trips can be freely downloaded from http://www.census.gov/population/www/cen2000/commuting/index.html.

The files were compiled from Census 2000 responses to the long-form (sample) questions on where individuals worked, and provide all the work destinations for people who live in each county. The data contain information on 34,116,820 commuters in 3,141 counties.

Demographic data containing the population and the geographic coordinates of the centroids of each county can be freely downloaded from https://www.census.gov/geo/www/gazetteer/places2k.html.

Our goal is to use the US commuting data to calculate the empirical distribution 
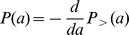
 and compare it to the theoretical predictions of the original radiation model, Eq. (3), and the radiation model with selection, Eq. (5).

We assume that the number of employment opportunities in every county, 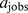, is proportional to the county’s population, 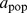, i.e. 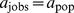, where 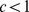 is the ratio between the average number of job offers considered by an individual (i.e. the ones known and of potential interest) over the population. Under this assumption, if we calculate the probability 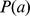 using the population instead of the job openings the resulting distribution is simply rescaled as 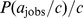.

From the census data we obtain the fraction of individuals who live in county 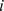 with population 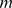 and work in county 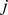 that lies beyond a circle containing a population 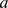 as 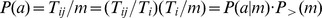, where 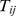 is the number of commuters from 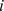 to 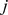, and 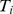 is the total number of commuters from 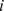 to all other counties. It follows that upon rescaling with 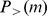, all the 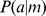 should collapse on the theoretical distribution 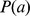. This is what we want to test in Fig. 2.

First, we divide the commuting fluxes in deciles according to the population of the origin county, 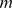. Then, for each set we calculate the distributions 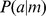 (Fig. 2a), and the rescaled distributions 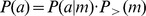 with 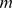 equal to the mean origin population of the counties in each set, and using the 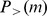 of Eq. (3) in Fig. 2b, and of Eq. (5) in Fig. 2c. The value of the parameter 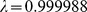 has been obtained by maximizing the likelihood that the observed fluxes are an outcome of the model. The discrepancy observed at very high 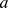 (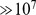) can be the result of boundary (finite-size) effects that become relevant at large populations, corresponding to long distances. Also, the fluctuations at very small 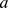 values are due to the resolution limit encountered when 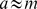. The parameter 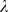 is close to 1 because in the comparison with data we consider populations instead of job offers and we assume that the two quantities are proportional, and consequently the fitting parameter we find is 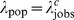, which is always close to 1 irrespective of 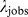 given that 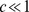.

### Analysis on Trips Extracted from a Mobile Phone Dataset

We use a set of anonymized billing records from a European mobile phone service provider [5], [31], [32]. The dataset contains the spatio-temporal information of the calls placed by 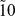
M anonymous users, specifying date, time and the cellular antenna (tower) that handled each call. Coupled with a dataset containing the locations (latitude and longitude) of cellular towers, we have the approximate location of the caller when placing the call. We analyze all call records collected during one day, and we define a trip when we observe two consecutive calls by the same user from two different towers. The type of mobility information obtained from the mobile phone data is radically different from that provided by the census data. In fact, the scope and method of the mobile phone data collection is complementary to the self-reported information of the census survey, and it offers the possibility to consider all trips, not only commuting (home-to-work) trips. Additionally, the mobility information that we extract from the mobile phone data is more detailed in both time and space. Indeed, we can observe trips of any duration, ranging from few minutes to several hours. In a similar manner, we can analyse trips on the much finer spatial resolution of cellular towers, whose average distance is 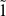
km, compared to the average size of counties, 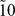
km. We are therefore including in the current analysis many more trips, obtaining a more complete picture of individual mobility.

In Figure 3 we use the trips obtained from the mobile phone data to provide a direct test of the models’ fundamental prediction, i.e. the specific functional form of the trips distribution 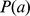. In the case of mobile phone data the trips’ destinations are determined by the particular purpose of the users when they start the trip. Therefore, the variable 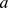 should now represent not only the number of job opportunities in a region, but rather the number of all possible venues that could be the destination of a trip, e.g. shopping centers, restaurants, schools, bars, etc. We therefore define the variable 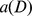, representing the number of possible points of interest in a circular region 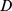 centered at a given cell tower, as the total number of calls placed from the towers in 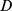, assuming that a location’s attractiveness is proportional to its call activity. We then calculate the empirical density distribution 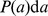, i.e. the fraction of trips to the towers between 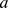 and 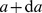, and we compare it to the various models’ theoretical predictions 
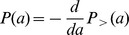
, with 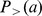 defined in Eqs. (5), (4), and (3), and whose parameters, 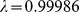 and 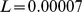, are obtained with least-squares fits. Moreover, we verified (plots not shown) that the result presented in Fig. 3 is stable with respect to other possible ways of defining a trip using the mobile phone data, e.g. between the two farthest locations visited by each user in 24 hours, or between the two most visited locations.
